# Integrated simultaneous fermentation–pervaporation for intensified bioethanol production and recovery in a corn stover cascade biorefinery

**DOI:** 10.1186/s40643-026-01152-y

**Published:** 2026-11-07

**Authors:** H. Candela, A. E. Illera, P. Barea, M. T. Sanz

**Affiliations:** Department of Biotechnology and Food Science, University of Burgos UBU, Plaza Misael Bañuelos S/N, 09001 Burgos, Spain

**Keywords:** Fermentation, Pervaporation, Bioethanol, Corn stover, Cascade biorefinery

## Abstract

Corn stover was valorized through an integrated cascade biorefinery combining subcritical water pretreatment, enzymatic hydrolysis, alcoholic fermentation, and pervaporation for intensified bioethanol production and recovery. Subcritical water treatment effectively solubilized the hemicelluloses while increasing cellulose accessibility in the solid residue. This enabled glucose yields of approximately 80% after 24 h of enzymatic hydrolysis. The resulting hydrolysate was then used in two strategies for the production and recovery of bioethanol. In the first one, the glucose-rich liquid was efficiently fermented by *Saccharomyces cerevisiae*, achieving complete glucose consumption within 15 h and an ethanol yield of 91%. Subsequently, ethanol was recovered through pervaporation, where silicone membranes were identified as the most suitable option due to their superior permeation performance. A novel integrated simultaneous fermentation–pervaporation (SFP) approach was developed as the second strategy to intensify bioethanol production and recovery by coupling both processes in a single operation. Operating the hybrid process at 38 °C substantially enhanced ethanol removal, allowing 98% of the produced ethanol to be recovered within only 15.3 h, compared with 60% recovery achieved by the sequential strategy. SFP at 38 °C achieved the highest overall yield, reaching 97% and demonstrated that integrating fermentation and pervaporation at this temperature effectively improved ethanol recovery and process productivity, providing a promising approach for sustainable lignocellulosic biorefineries.

**Graphical abstract:**

**Figure:**
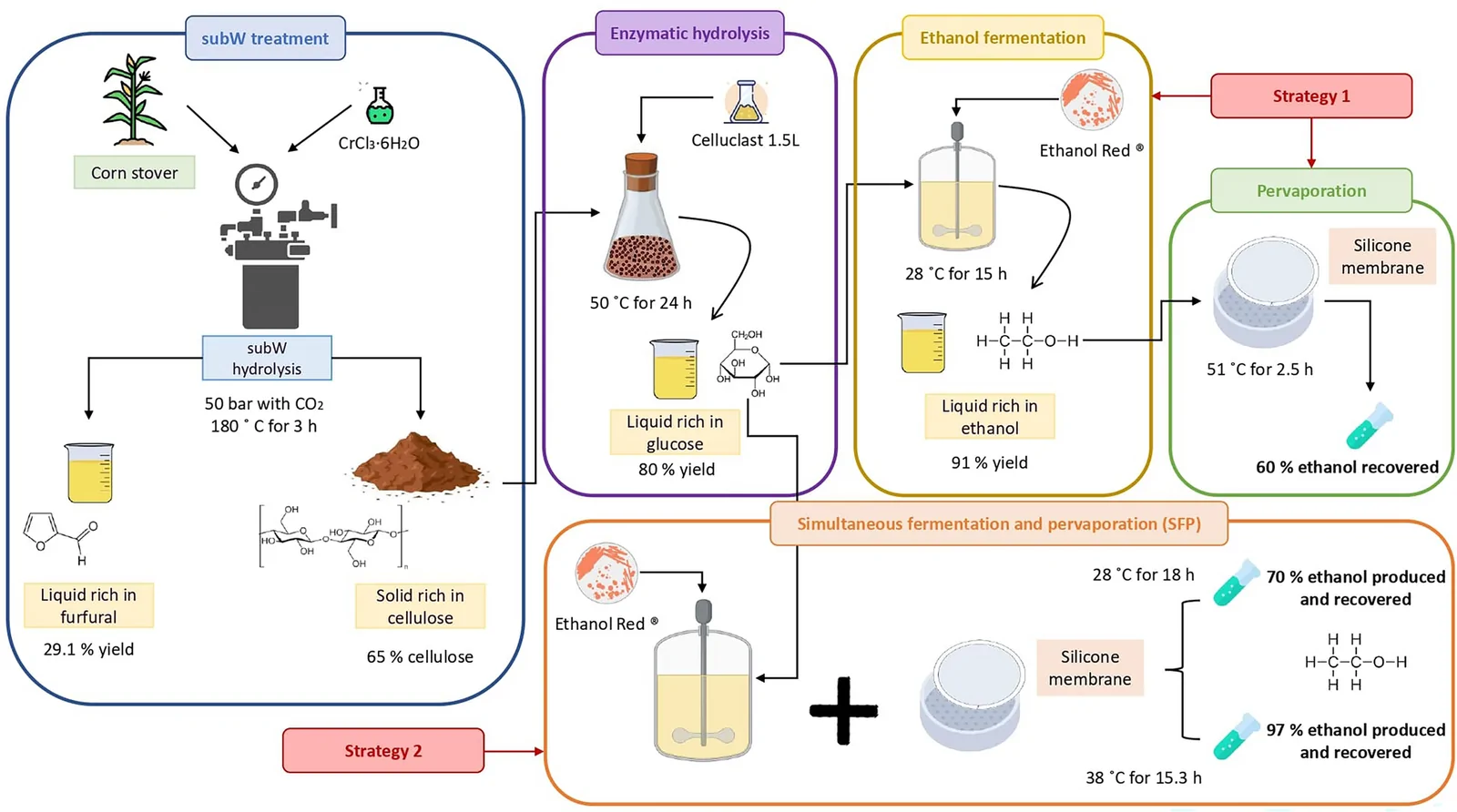

**Supplementary Information:**

The online version contains supplementary material available at https://doi.org/10.1186/s40643-026-01152-y.

## Introduction

The transition towards a low-carbon economy has accelerated the development of sustainable biorefineries capable of converting renewable biomass into fuels, chemicals and value-added products (Alonso-Riaño et al. 2023). Among the available feedstocks, lignocellulosic agricultural residues are particularly attractive because of their abundance, low cost and limited competition with food resources. Among agricultural residues, corn stover stands out as the second most promising agricultural waste in the European Union bioeconomy, only after wheat straw (Azlan et al. 2022). The residue-to-crop ratio average for corn stover is 1.13, due to the fact that almost 50% of the external corn plant is stover. Given this ratio and its chemical composition (17 ± 3% lignin, 37 ± 3% cellulose, and 26 ± 4% hemicellulose), corn stover stands out as a promising biomass to produce environmentally friendly energy and products (Thorenz et al. 2018).

Bioethanol has been established as a sustainable renewable resource due to its ability to significantly cut down on carbon dioxide emissions and reduce dependence on fossil fuels, through relatively simple fermentation processes (Melendez et al. 2022). However, the composition of lignocellulosic biomass presents significant challenges for bioethanol production. Lignin is associated with cellulose and hemicellulose, creating a cell wall that is extremely resistant to enzymatic degradation, required to enable glucose fermentation (Gao et al. 2018; Luo et al. 2019).

Biomass components can be hydrolyzed through enzymatic, microbial and thermochemical treatments (Singh et al. 2022). Among the last ones, hydrothermal treatments such as subcritical water (subW) are outstanding. SubW is water that remains liquid in the temperature range of 100–374 °C, under pressure. It presents special physicochemical properties, such as higher ionic product and lower dielectric constant than under ambient conditions, promoting hydrolysis reactions and greater solubility of organic compounds in an aqueous environment (Illera et al. 2024). By tailoring operating conditions, subW provides a powerful fractionation tool, enabling the selective extraction of biomass components that can be directed towards the production of valuable chemicals, while avoiding the use of organic solvents.

Despite the maturity of bioethanol fermentation technologies, downstream recovery remains one of the most energy-intensive stages of the overall process, particularly when conventional distillation is employed. Among membrane-based separation technologies, pervaporation has attracted considerable attention because it enables selective ethanol recovery at lower energy demand than conventional distillation while being readily integrated with fermentation processes (Peng et al. 2021). Pervaporation (PV) was selected as the bioethanol recovery technology in this work. It combines permeation and evaporation by bringing a liquid feed stream into contact with one side of a porous or non-porous membrane while applying a vacuum that generates a permeate vapor stream on the other side of the membrane (Vane 2008). PV has proven to be an effective technology for the separation of ethanol mixtures in water, as well as in fermentation broths or lignocellulosic hydrolysates. Bello et al. (2014) performed PV recovery of ethanol from banana fermentation broth, and more recently, Imad and Castro-Muñoz (2023) provided a comprehensive review of the development of pervaporation ethanol-separation membranes, covering polymeric, inorganic, and mixed-matrix membranes.

All the above-mentioned methodologies were combined in a cascade biorefinery approach for the total valorization of corn stover with two different strategies involving the production and recovery stages. The first two steps were common to both strategies. First, corn stover was treated with subW, producing cellulose-enriched solid and liquid rich in furfural. During subW treatments under mild conditions (160–210 °C), most of the hemicellulose fraction is solubilized and dehydrated to furfural, while the remaining solid is enriched in cellulose and lignin (Illera et al. 2024). Furfural is considered by the US Department of Energy (DOE) one of the top 30 platform chemicals that could be produced from biomass (Lee and Wu 2021). The second step was the enzymatic hydrolysis of the generated solid, enriched in cellulose, to enhance the fermentation stage, by releasing glucose monomers (saccharification) (Alonso-Riaño et al. 2022). The third step was the production of bioethanol through the fermentation of the hydrolysate rich in glucose with Ethanol Red®, and the fourth and final step in the cascade process was bioethanol recovery through pervaporation.

The conventional sequential strategy (Strategy 1) comprises four consecutive steps (subW pretreatment, enzymatic hydrolysis, fermentation and pervaporation), whereas the proposed novel hybrid strategy (Strategy 2) integrates fermentation and pervaporation into a single simultaneous operation following subW pretreatment and saccharification.

Some research has been conducted on continuous coupling of fermentation and pervaporation (Fan et al. 2014) using a feed reservoir. However, as far as reported in the literature, the simultaneous integration of fermentation and pervaporation (Strategy 2) without continuous operation and without a separate fermentation vessel has not yet been reported. This study introduces such a novel approach, aiming to shorten operation time through a one-pot process. Together with the subcritical water pretreatment, the proposed integrated process provides a sustainable and intensified strategy for the cascade valorization of corn stover into furfural and bioethanol.

## Experimental section

### Materials

#### Raw material

The raw material used in this work was obtained from corn harvest in Palencia, Spain. Corn stover (leaves and stalks), was separated from corn cobs and then dried at room temperature until constant moisture. They were then ground to a size of less than 2 mm before their use. Moisture was determined with the Ohaus MB120 Moisture Analyzer and biomass characterization was performed according to National Renewable Energy Laboratory (NREL) protocols (Sluiter et al. 2008), as later described.

#### Chemicals and other materials

Glucose (99%) and CrCl_3_·6H_2_O (98%) were purchased from Sigma-Aldrich. *Celluclast* 1.5 L was kindly provided by Novozymes A/S. Meat peptone, absolute ethanol (99.8%) and (NH_4_)_2_SO_4_ (99%) were purchased from VWR. Bacteriological agar, yeast extract and malt extract were purchased from Scharlab. (NH_4_)_2_HPO_4_ and MgSO_4_⋅7H_2_O (99.5%) were purchased from PanReac. *Saccharomyces cerevisiae* (Ethanol Red®) was kindly provided by Lesaffre Advanced Fermentations (Marcq-en-Baroeul, France). Two different organophilic dense membranes were kindly provided by Helmholtz-Zentrum Hereon (Germany) and tested in this study. The difference remained in the membranes active layer, one of them was based on polyoctylmethyl siloxane (POMS), and the other one on silicone.

### Subcritical water treatment

In order to achieve a total valorization of corn stover, a cascade process was proposed. First, corn stover was subjected to a subcritical water (subW) hydrolysis to produce furfural and generate a glucose-enriched solid residue. This process had been previously optimized in our research group, and consisted in a subW treatment under mild conditions (corn stover 5% w/v, 0.2 L, 180 ˚C, 3 h of isothermal treatment at 50 bar with CO_2_ pressurization), using CrCl_3_·6H_2_O (2% w/w of xylose in corn stover) as catalyst. The equipment used was similar to that described by Illera et al. (2024). After this first step, two fractions were obtained. A liquid fraction, containing furfural produced by the hemicellulose from corn stover, and a solid fraction enriched in cellulose. This solid fraction was the one used for the following steps of the cascade approach.

### Enzymatic hydrolysis

To obtain fermentable monosaccharides from the corn stover cellulose fraction, an enzymatic hydrolysis (HE) of the pretreated solid was carried out. For this, *Celluclast* 1.5 L, a cellulolytic cocktail which enzymatic activity consists of the hydrolysis of (1,4)-beta-D-glycosidic bonds in cellulose and other beta-D-glucans in free glucose monomers, was used. It is a cocktail of liquid cellulases with a density of 1.2 g/mL and an endoglucanase activity of 700 units/g. Its activity in filter paper units (FPU) was determined according to the NREL standard procedure as 53.7 FPU/mL and an activity of 45 FPU/g was used for hydrolysis (Alonso-Riaño et al. 2022). HE was performed using the corn stover subW-treated solid at 8% w/v in 50 mM citrate buffer (pH 4.8). The hydrolysis was carried out at a constant temperature of 50 °C in a 500 mL jacketed reactor with magnetic stirring. Samples were collected at different times to determine the kinetics of the reaction until the glucose content was constant in the hydrolysate. To stop the hydrolysis in the collected and final samples, the enzyme was inactivated by heat treatment at 95 °C for 5 min. After centrifugation, both solid and liquid fractions were stored under refrigeration for next steps. Raw corn stover was also enzymatically hydrolyzed to test the effect of the subW pretreatment on cellulose accessibility. The HE process was evaluated by the glucose conversion yield, which relates the amount of glucose released by the enzymes and the maximum potential glucose amount obtained from the solid (Eq. 1):

1 $${Yield}_{glucose} \left(\%\right)= \frac{enzymatic \, hidrolysate \, glucose (g)}{solid \,residue \,glucose (g)}*100$$

### Alcoholic fermentation

The glucose-enriched liquid fraction obtained from HE was used as the substrate to produce bioethanol. For this purpose, Ethanol Red® yeast, a *Saccharomyces cerevisiae* strain developed for industrial ethanol production, was selected. The strain was incubated at 28 °C for 48 h and maintained at 4 °C in a solid medium consisting of 10 g/L glucose, 5 g/L peptone, 3 g/L malt extract, 3 g/L yeast extract and 20 g/L agar. Pre-inoculum was prepared by transferring a yeast colony from the solid medium to 10 mL of sterilized liquid medium (same formulation as the solid medium except for the absence of agar) and incubated in agitation at 180 rpm at 28 °C for 24 h. Inoculum was prepared by transferring the pre-inoculum to 40 mL of liquid medium and incubation at 28 °C for 14 h. Optical density of the biomass (inoculum) was measured at λ = 620 nm (UVmini-1240, Shimadzu, Tokyo, Japan) and the biomass concentration was determined using a calibration curve.

The fermentation reaction required enzymatic hydrolysate (85% v/v), supplementation solution (5%) and an inoculum volume which ensured a concentration of 0.2 g/L biomass at the start of fermentation, adjusted with a 0.9% v/v NaCl solution. The supplementation solution (80 g/L (NH_4_)_2_HPO_4_, 40 g/L (NH_4_)_2_SO_4_, 20 g/L MgSO_4_⋅7H_2_O and 100 g/L yeast extract; pH of 5.5) was added to provide essential nutrients and buffering capacity for fermentation.

Fermentation was carried out at 28 °C and 180 rpm and was stopped after 24 h. Samples were periodically collected to determine the concentration of remaining sugars, produced ethanol, as well as the optical density of the biomass.

The fermentation process was evaluated through ethanol production yield (Eq. 2).

2 $${Yield}_{ethanol} \left(\%\right)= \frac{g ethanol \,produced}{g {glucose}_{broth}*0.511}*100$$

where the theoretical conversion factor of 0.511 corresponds to the maximum stoichiometric yield from glucose fermentation (C_6_H_12_O_6_ → 2 C_2_H_5_OH + 2 CO_2_).

### Pervaporation

Pervaporation was chosen as the technique to separate ethanol from the fermentation broth. Two organophilic polymeric membranes were studied for ethanol recovery: POMS membrane and silicone membrane.

**Fig. 1:**
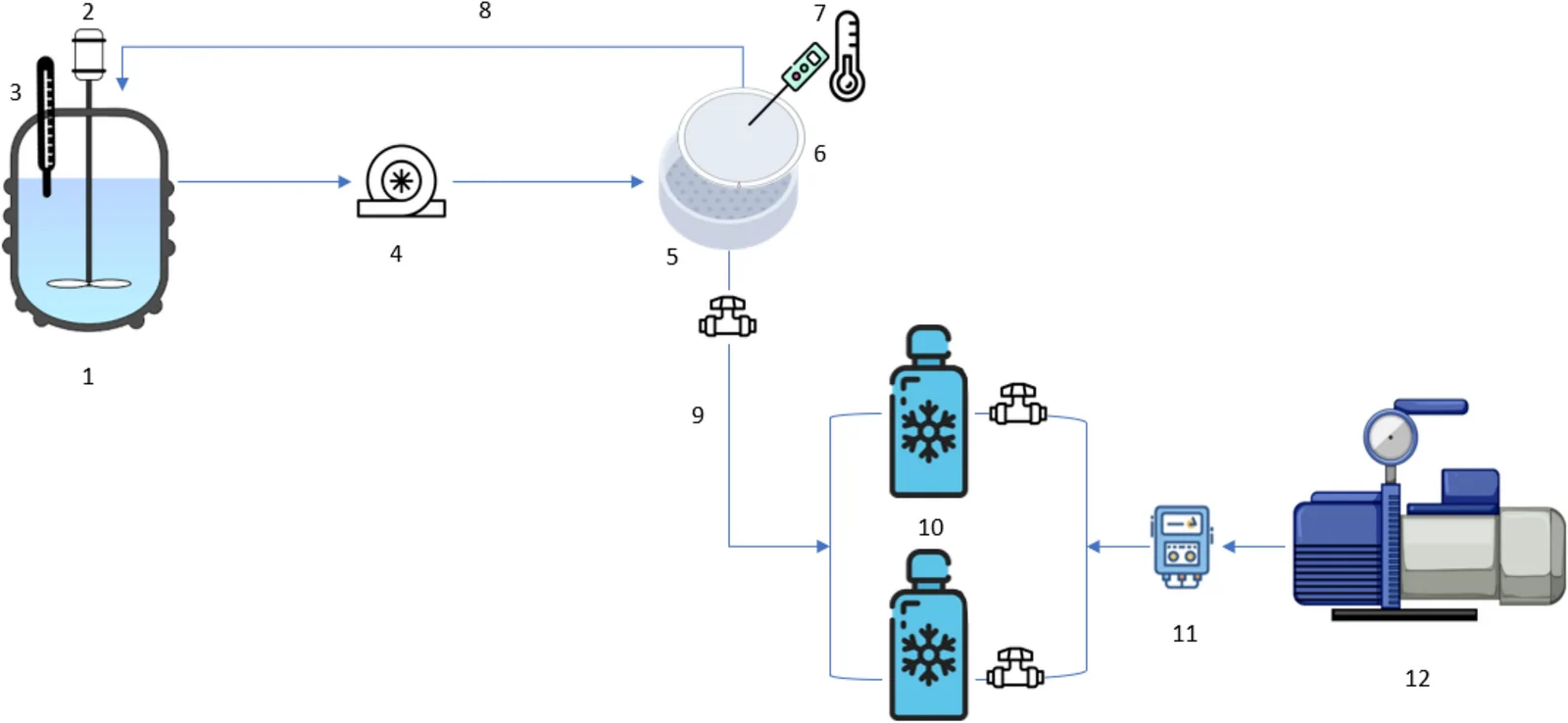
Diagram of the pervaporation equipment. 1. Jacketed feed tank; 2. Stirrer; 3. Thermostat; 4. Peristaltic pump; 5. Pervaporation cell; 6. Membrane; 7. Thermostat; 8. Retentate; 9. Permeate; 10. Glass traps in liquid N_2_; 11. Vacuum controller; 12. Vacuum pump

The pervaporation equipment consisted of a jacketed feed tank with a stirrer (IKA® RW 16 basic overhead stirrer), a peristaltic pump (qdos30 Watson Marlow) and a stainless-steel pervaporation cell for membranes with an effective area of 170 cm^2^ (Sulzer Chemtech®). This system provided a radial feed flow over the membrane surface with a flow rate set at 102 kg/h, which was high enough to avoid concentration polarization. A coil was attached to the tank as a coolant for condensation of possible vapors. The feed solutions were pumped from the jacketed tank to the membrane module. In the pervaporation cell, ethanol diffused selectively through the membrane and was transferred to the permeate side in vapor phase, while the remaining components were recirculated to the feed tank. In the permeate side, the system was connected to 2 parallel glass traps immersed in liquid nitrogen in which the vapor stream was condensed. The permeate pressure was kept constant at 3 mbar using a vacuum pump (Edwards RV12) and a vacuum controller (CVC-3000). As feed solutions, distilled water, ethanol solutions, or fermentation broth were used in the different pervaporation studies. The pervaporation assembly was described in Fig. 1.

#### Pervaporation evaluation

Pervaporation performance was previously described by Alonso-Riaño et al. (2023). The total permeate flux ${(J}_{total})$ was evaluated considering the amount of permeate, time and membrane area (Eq. 3).

3 $${J}_{total}= \frac{permeate (g)}{time \left(h\right) *membrane area ({m}^{2)}}$$

The ethanol flux was calculated from the total permeation flux and the ethanol mass fraction of the permeate according to Eq. (4).

4 $${J}_{ethanol}{=J}_{total}{* w}_{ethanol}$$

The separation yield of the pervaporation membranes was tested by the enrichment factor (β), defined as the ratio of its concentration in the permeate to the feed (Eq. 5):

5 $$\beta {=w}_{ethanol, permeate}{ / w}_{ethanol, feed}$$

Finally, the yield of the membranes was evaluated using the pervaporation separation index (PSI), which relates the permeation flux and the feed separation factor (Eq. 6):

6 $$PSI{=J}_{total}* \beta $$

The pervaporation process was evaluated through ethanol recovery yield (Eq. 7).

7 $${Yield}_{ethanol recovery} \left(\%\right)= \frac{g ethanol produced}{g ethanol recovered}*100$$

#### Effect of temperature and membrane material

First, the effectiveness of the selected hydrophobic membranes for ethanol separation and the effect of temperature on the permeate flux were simultaneously studied. For this purpose, the working temperature varied in a range between 30 and 54 °C with a vacuum pressure of 3 mbar. This experiment was carried out with both POMS and silicone membranes, using first water as feed and then a 15 g/L ethanol solution. All experiments were carried out in a 5 L jacketed reactor with an initial volume of 3 L. The effect of temperature on the permeate flux was described using the Arrhenius equation (Eq. 8):

8 $$ J = Jo* e^{{\left( {{\raise0.7ex\hbox{${ - Ea}$} \!\mathord{\left/ {\vphantom {{ - Ea} {R \cdot T}}}\right.\kern-0pt} \!\lower0.7ex\hbox{${R \cdot T}$}}} \right)}} $$

where *J* is the permeate flux (g·min^−1^·m^−2^), *Ea* is the apparent activation energy of permeate (kJ/mol), *R* is the ideal gas constant, Jo is the pre-exponential factor (g·min^−1^·m^−2^) and temperature is the absolute temperature (K).

#### Effect of ethanol concentration

Once the most selective membrane towards ethanol and the optimum temperature (54 °C) were identified, the effect of ethanol concentration in the pervaporation performance was studied. 3 L ethanol solutions were used as feed in the 5 L jacketed reactor, with varying ethanol concentrations from 1.5 to 45 g/L.

### Ethanol recovery from fermentation broth

Following the optimization of pervaporation with ethanol solutions, best conditions were selected for the recovery of ethanol from real fermentation broth using two different strategies. For those experiments, volume feed was reduced to 0.26 L, and they were performed in a 0.5 L jacketed tank.

In order to compare the performance of the two strategies, the yield of both the fermentation and pervaporation stages was calculated together evaluating the overall yield (Eq. 9).

9 $${Yield}_{fermentation+pervaporation} \left(\%\right)= \frac{g ethanol recovered}{ g glucose broth*0.511}*100$$

where the recovered ethanol is evaluated over the theoretical ethanol production taking into account the initial glucose in the broth.

#### Fermentation followed by pervaporation (Strategy 1)

Fermentation of glucose to ethanol was first performed in a separate reactor, as described in Sect. "Alcoholic fermentation". Afterwards, the fermentation broth was transferred to the jacketed reactor for the recovery of ethanol by pervaporation. The feed tank temperature was set to 54 °C and the temperature in the PV cell was constant during the process with a value of 51 ± 2 °C. The pervaporation was stopped when the ethanol concentration was below 1 g/L in the feed (3 h process). The permeate and feed samples were periodically collected and analyzed by HPLC.

#### Simultaneous fermentation and pervaporation (SFP) (Strategy 2)

**Fig. 2:**
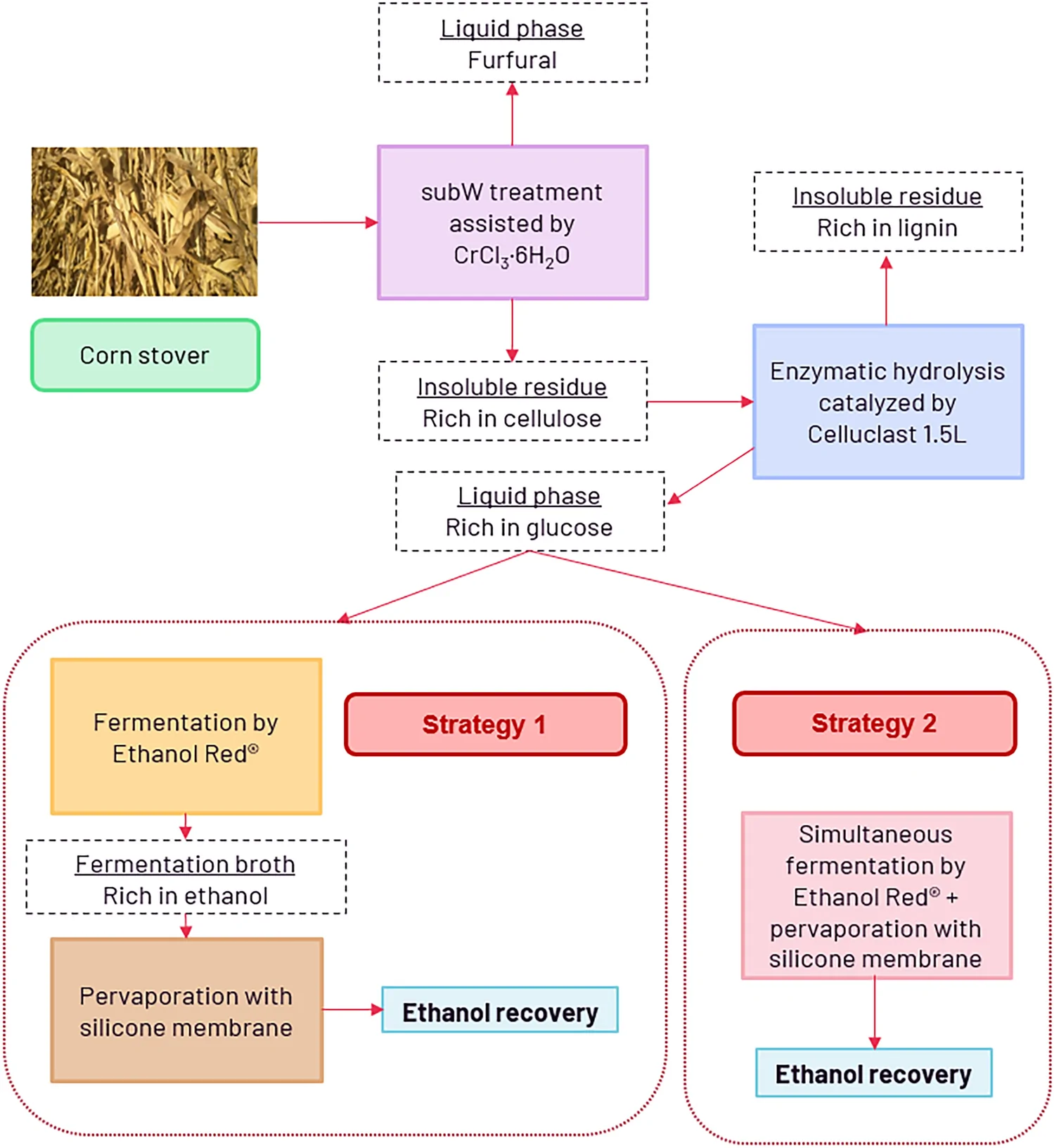
Flow chart of the methodological process

In the second strategy, a new approach consisting of a simultaneous fermentation and pervaporation (SFP) process was proposed to reduce operating time and simplify the process in a one-pot approach (Fig. 2). First, the fermentation broth (260 mL) remained in the tank (no transfer across the membrane) at 28 °C for 4 h, as the necessary time for the adaptation process of the microorganism. After that, the fermentation broth started to be recirculated (flow rate set at 102 kg/h) and a vacuum up to 3 mbar was applied. From 4 h onwards, the permeate was collected in a trap immersed in N_2_. The permeate and feed samples were periodically collected to follow the process performance. The process was stopped when the glucose was depleted (no more ethanol could be produced by fermentation) and the ethanol concentration in the feed tank was less than 1 g/L.

##### Optimization of SFP. Effect of temperature in fermentation

The recommended fermentation temperature (28 °C) and the optimum pervaporation temperature (54 °C) are far apart. In this regard, it was proposed to find a higher fermentation temperature to improve ethanol diffusion through the membrane and achieve effective SFP in a single step, while ensuring the survival and activity of the microorganism. Fermentation was tested at temperatures ranging from 28 °C to 43 °C, following the Alcoholic fermentation" section methodology.

##### SFP at higher temperature

The highest temperature at which the yeast properly functioned (38 °C) was selected, and SFP was carried out at this temperature. With the optimum working parameters, the enzymatic hydrolysate from pre-treated corn stover was used as substrate for this final testing, as a real approach towards the cascade valorization of corn stover.

### Analytical methods

#### Determination of structural components. Lignin and carbohydrates

Biomass characterization was performed according to NREL protocols (Sluiter et al. 2008). This procedure uses a two-step acid hydrolysis in which a 72% w/w sulfuric acid solution is used for primary hydrolysis, followed by a secondary hydrolysis at high temperature (autoclave at 121 °C for 60 min). During hydrolysis, the lignin is fractionated into acid-insoluble material and acid-soluble material, which are measured separately. The acid-insoluble material is filtered under vacuum and measured gravimetrically after 24 h muffle incineration. Acid soluble lignin is measured spectrophotometrically (*λ* = 320 nm). Structural carbohydrates are hydrolyzed into monomeric sugars and quantified by HPLC. Characterization of the solids after the subW treatment and after enzymatic hydrolysis, as well as of the untreated biomass, was performed.

#### pH

pH values were measured in all samples using a GLP 21 pH meter (Crison Instruments S.A.).

#### High performance liquid chromatography (HPLC)

Quantification of the hydrolyzed, fermented and permeated compounds (furfural, glucose, xylose, arabinose and ethanol) was performed by high performance liquid chromatography (HPLC) using a Bio-Rad Aminex-HPX-87H column (300×7.8 mm, Bio-Rad), coupled to a variable wavelength detector (VWD) and a refractive index detector (RID) using a mobile phase consisting of 0.005 M sulfuric acid. The column and detector were maintained at 40 °C.

### Statistical analysis

The structural composition of all samples was analyzed in duplicate. subW treatment was performed in triplicate and HE in duplicate for each fermentation test using HE hydrolysate. All fermentation and pervaporation processes, both involving standard solution preparations or HE hydrolysates have been performed in duplicate. Statgraphics v.19 was used for the statistical analysis of the data through ANOVA and LSD (Least significant difference) tests where needed.

## Results and discussion

The cascade process results are described in this section, evaluating the transformation of raw corn stover through the different steps until the final ethanol recovery by pervaporation strategies. For a better tracking of the whole process, Table S1 describes in detail the transformation of 1 kg of dry raw corn stover through each of the described stages, indicating the desire product in each of them as well as yield, concentration and product amount.

### Biomass characterization

The structural composition of corn stover presented a high polysaccharide content (42% cellulose and 30.3% hemicellulose), making it an ideal candidate for the production of different building blocks from sugars (Table 1).

**Table 1:** Chemical composition in dry weight percentage of raw corn stover, subW solid residue and subW-treated corn stover after enzymatic hydrolysis (HE solid residue)

Components	Raw corn stover	subW solid residue	HE solid residue
Lignin	25.7 ± 0.5^a^	32 ± 2^b^	66 ± 3^c^
Soluble lignin	8.3 ± 0.2^c^	3.0 ± 0.1^a^	4.0 ± 0.1^b^
Insoluble lignin	17.4 ± 0.4^a^	29 ± 2^b^	62 ± 3^c^
Cellulose	42 ± 1^b^	66 ± 3^c^	16.9 ± 0.1^a^
Hemicellulose	30.3 ± 0.8^c^	4.1 ± 0.1^b^	2.6 ± 0.1^a^
Xylose	25.8 ± 0.7^c^	4.1 ± 0.1^b^	2.6 ± 0.1^a^
Arabinose	4.5 ± 0.1	n.d	n.d
Ashes	4.3 ± 0.1^a^	4.3 ± 0.1^a^	9 ± 3^b^

Different letters in the same row indicate significant differences between samples for the analyzed component (LSD test, 95% confidence). n.d.: non detected

### Subcritical water treatment

After subcritical water treatment, the liquid fraction contained 3.2 ± 0.1 g/L of furfural, corresponding to a furfural yield of 29.1 ± 1.2%. Furfural yield was calculated as the moles of furfural formed from the moles of pentose sugars available in the corn stover, as they show a one-to-one molar conversion (Illera et al. 2024). The treated subW solid exhibited a significant reduction in hemicellulose content, from 30.3 to 4.1% (Table 1), as a result of hemicellulose hydrolysis into soluble sugars followed by their thermochemical dehydration to furfural. Consequently, the cellulose content increased from 42 to 66%, indicating that most of the cellulose remained in the solid fraction after subcritical water treatment. These results confirm that the selected subcritical water conditions efficiently fractionated corn stover, simultaneously enabling furfural production from the hemicellulosic fraction while preserving cellulose for subsequent enzymatic conversion.

### Enzymatic hydrolysis

**Fig. 3:**
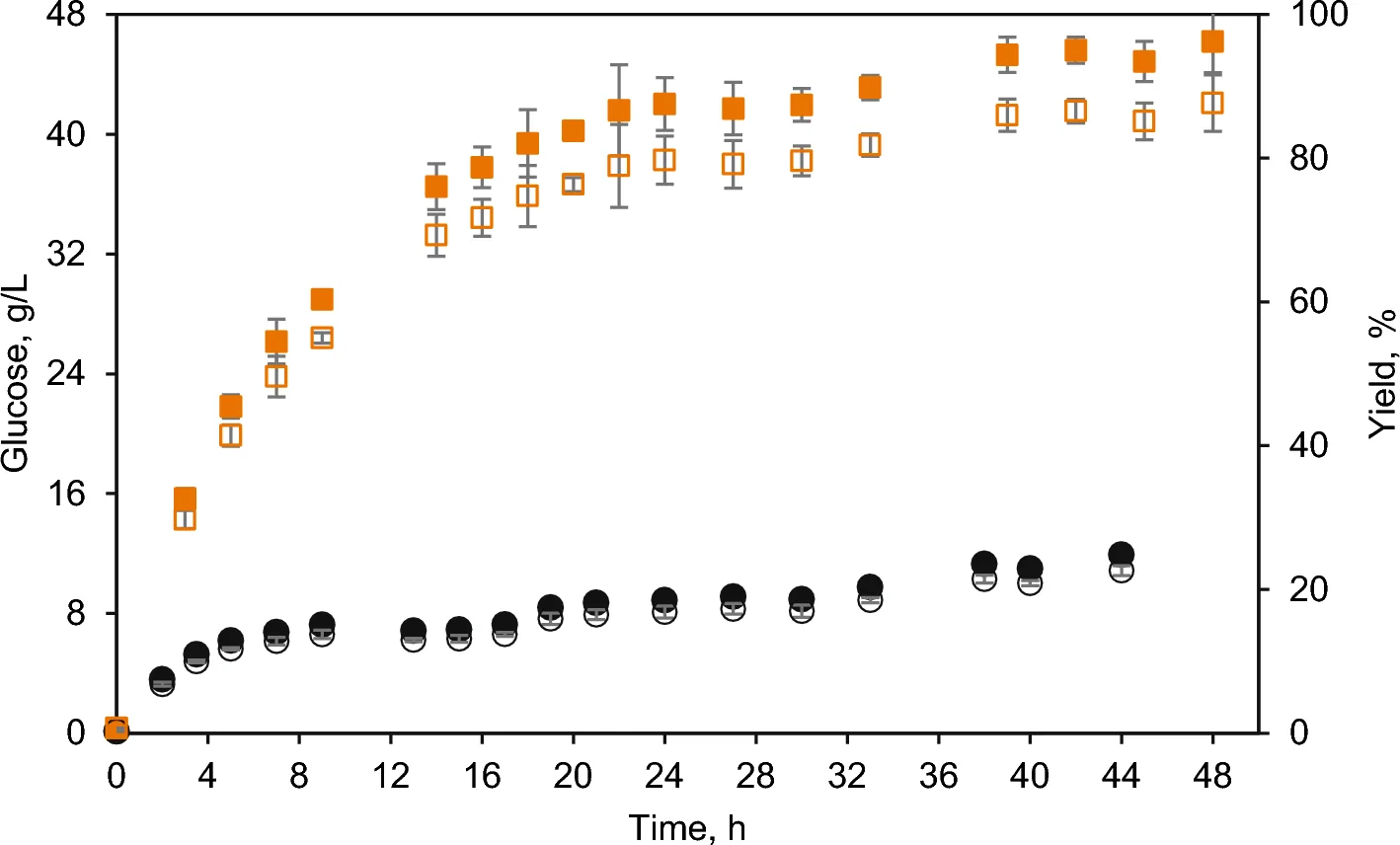
Time course of enzymatic hydrolysis of () raw and () subW-treated corn stover (8% w/v). Open symbols (, ) represent hydrolysis yield (%), whereas filled symbols (, ) represent glucose concentration (g glucose/L of hydrolysate).

To evaluate the effect of subcritical water pretreatment on cellulose accessibility, enzymatic hydrolysis (HE) was performed on both raw and subW-treated corn stover (Fig. 3). The conversion to glucose was almost 5 times higher in subW-treated corn stover (42 ± 3 g/L glucose after 24 h, 80 ± 3% yield) compared to raw corn stover (9 g/L glucose after 24 h; 17% yield), proving the importance of the subW pretreatment role. This behaviour agrees with previous studies reporting that residual hemicellulose can hinder enzymatic cellulose hydrolysis by limiting enzyme accessibility (Alonso-Riaño et al. 2022). Most of the glucose was released during the first 12–16 h of hydrolysis and only a slight increase was observed between 24 and 48 h (42 to 46 g/L), indicating that enzymatic hydrolysis had nearly reached completion after 24 h and extending the process would not provide significant improvements in glucose production.

As shown in Table 1, after HE the main component was lignin, confirming the efficient conversion of cellulose into soluble glucose, with only 17% of the initial cellulose remaining in the solid fraction. Under the evaluated conditions, the optimum HE performance was achieved after 24 h at 50 °C using an 8% (w/v) pretreated biomass loading.

### Alcoholic fermentation

**Fig. 4:**
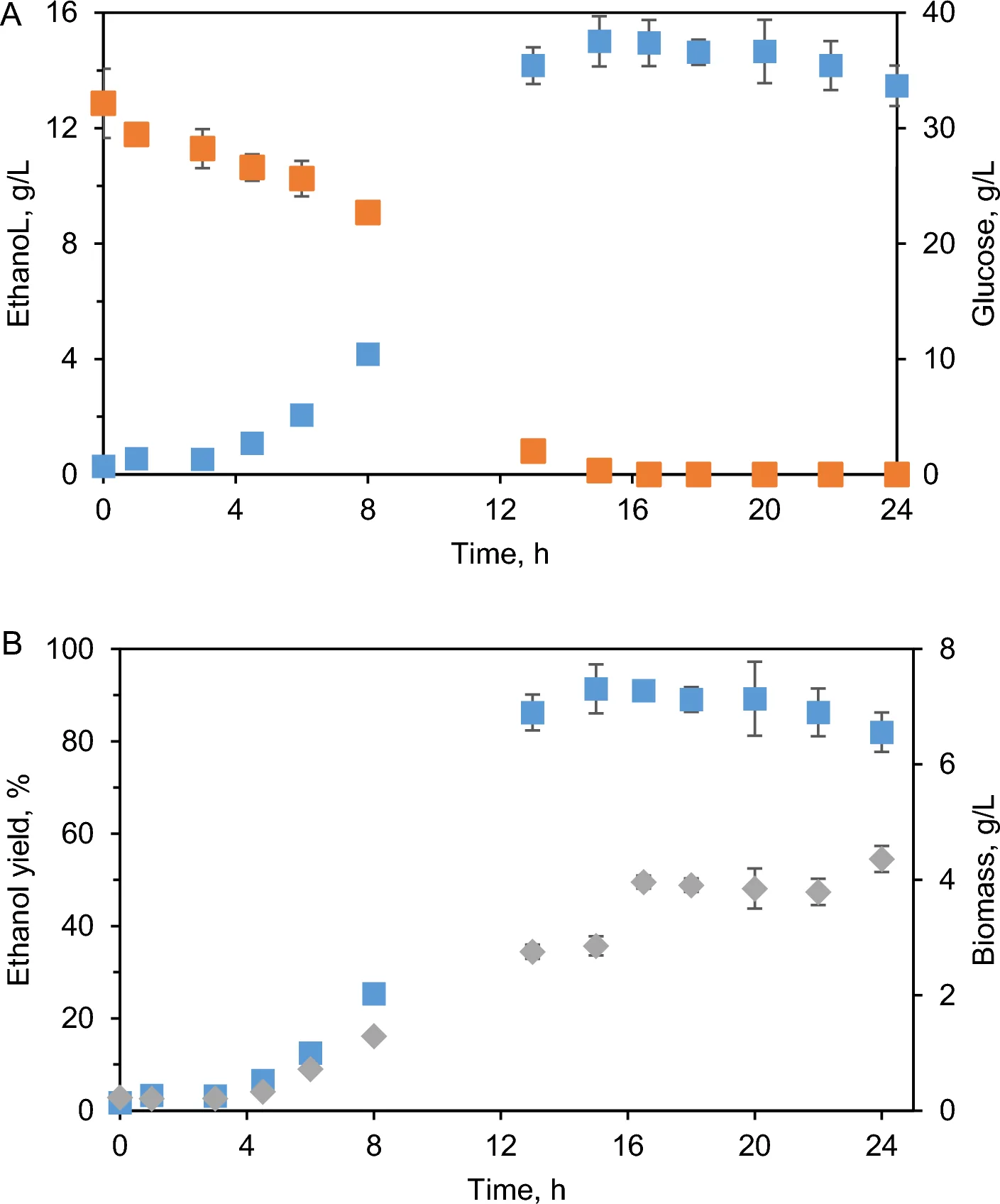
Fermentation kinetics of the enzymatic hydrolysate obtained from subW treated corn stover. **A** () Glucose and () ethanol concentrations (g/L). **B** () Biomass concentration (g/L) and () ethanol yield (%)

Bioethanol production was evaluated by fermenting the enzymatic hydrolysate obtained from the subW-treated solid using Ethanol Red®. During the whole fermentation, the pH remained unchanged (pH = 4.5 ± 0.2). Glucose consumption, ethanol production and biomass growth are shown in Fig. 4. At the beginning of the fermentation, the glucose concentration was 32 ± 3 g/L (Fig. 4A). This decrease in glucose concentration compared to the one measured after enzymatic hydrolysis, with a concentration of 42 ± 3 g/L, corresponds to its dilution by the addition of the inoculum and the supplementation solution. The same dilution effect took place when later testing the SFP strategy.

Glucose was rapidly consumed during fermentation, decreasing to 2 g/L after 13 h and to only 0.3 g/L after 15 h (99% consumption), with complete depletion achieved after 16.5 h.

Ethanol concentration increased steadily during glucose consumption, reaching a maximum of 15.0 g/L after 15 h (Fig. 4A). Thereafter, a slight decrease was observed, reaching 13.5 g/L after 24 h. This behaviour could be attributed to ethanol reassimilation by the yeast, since biomass continued to increase from 2.9 to 4.4 g/L after glucose depletion (Fig. 4B).

Previous studies have shown that *S. cerevisiae* can reassimilate ethanol after glucose depletion and may also consume other carbon sources to sustain cell growth (Snoek et al. 2016; Branco et al. 2020). In the present work, xylose concentration remained constant throughout fermentation, suggesting that biomass growth after glucose depletion was mainly associated with ethanol reassimilation and possibly with the consumption of other metabolites not quantified, as previously reported by (Alonso-Riaño et al. 2022). The maximum ethanol production yield observed (91 ± 1%) was after 15 h (Fig. 4B), demonstrating an efficient conversion of glucose into ethanol under the selected operating conditions. This value is higher than those reported for several lignocellulosic feedstocks subjected to sequential hydrolysis and fermentation, as in a study where subW-pretreated brewer’s spent grain was used, and the maximum ethanol yield obtained was 83% after 48 h (Alonso-Riaño et al. 2022). Tsegaye et al. (2019) achieved an ethanol yield of 68.2% in a 96 h wheat straw fermentation after a microwave-assisted NaOH pretreatment. Chen et al. (2021) reported a slightly higher ethanol yield (93%) that required 32 h of fermentation, whereas a comparable yield (91 ± 1%) was achieved in the present work after only 15 h.

### Pervaporation

#### Effect of temperature and membrane material

**Fig. 5:**
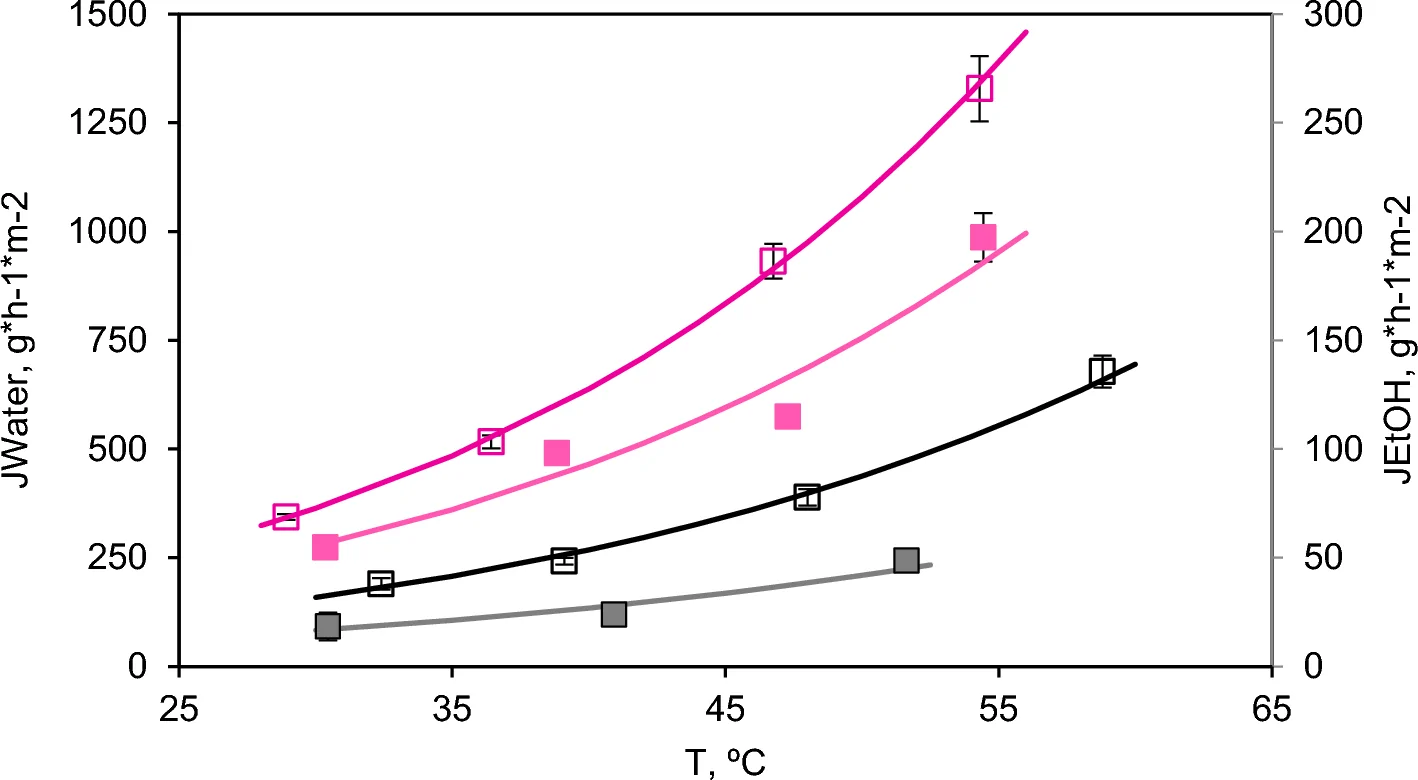
Permeation flux of (, ) distilled water and (, ) 15 g/L ethanol solution as a function of temperature using the (, ) silicone membrane and (, ) POMS membrane. Lines correspond to the Arrhenius equation

First, the effect of temperature was studied for the silicone and POMS membranes. Figure 5 shows the permeate flux for both membranes working with pure water or ethanol (15 g/L) as feed. Permeation flux increased with temperature following an Arrhenius-type behaviour, indicating that mass transport through both membranes was thermally activated. Higher temperature increases the movement of the polymeric chains of the membrane improving molecules diffusion. Both for water and the ethanol solution, it was observed that the flux achieved with the silicone membrane was much higher than that of the POMS membrane at all temperatures studied. The difference in the permeate fluxes of both membranes was due to the physicochemical characteristics of the material of the selective layer. Moreover, the permeate of the silicone membrane generated a higher ethanol flux than the POMS membrane at any T, making this membrane more suitable for ethanol recovery from fermentation broths.

Ea_Water_ and Ea_Ethanol_ of both membranes was evaluated, being Ea_Ethanol_ 41 and 38 kJ/mol for silicone and POMS membrane, respectively, and Ea_Water_ 44 and 41 kJ/mol for silicone and POMS membrane, respectively. Although the silicone membrane exhibited slightly higher activation energies, the differences were relatively small, suggesting that the superior permeation performance mainly arises from its higher intrinsic permeability rather than from a markedly stronger temperature dependence.

**Fig. 6:**
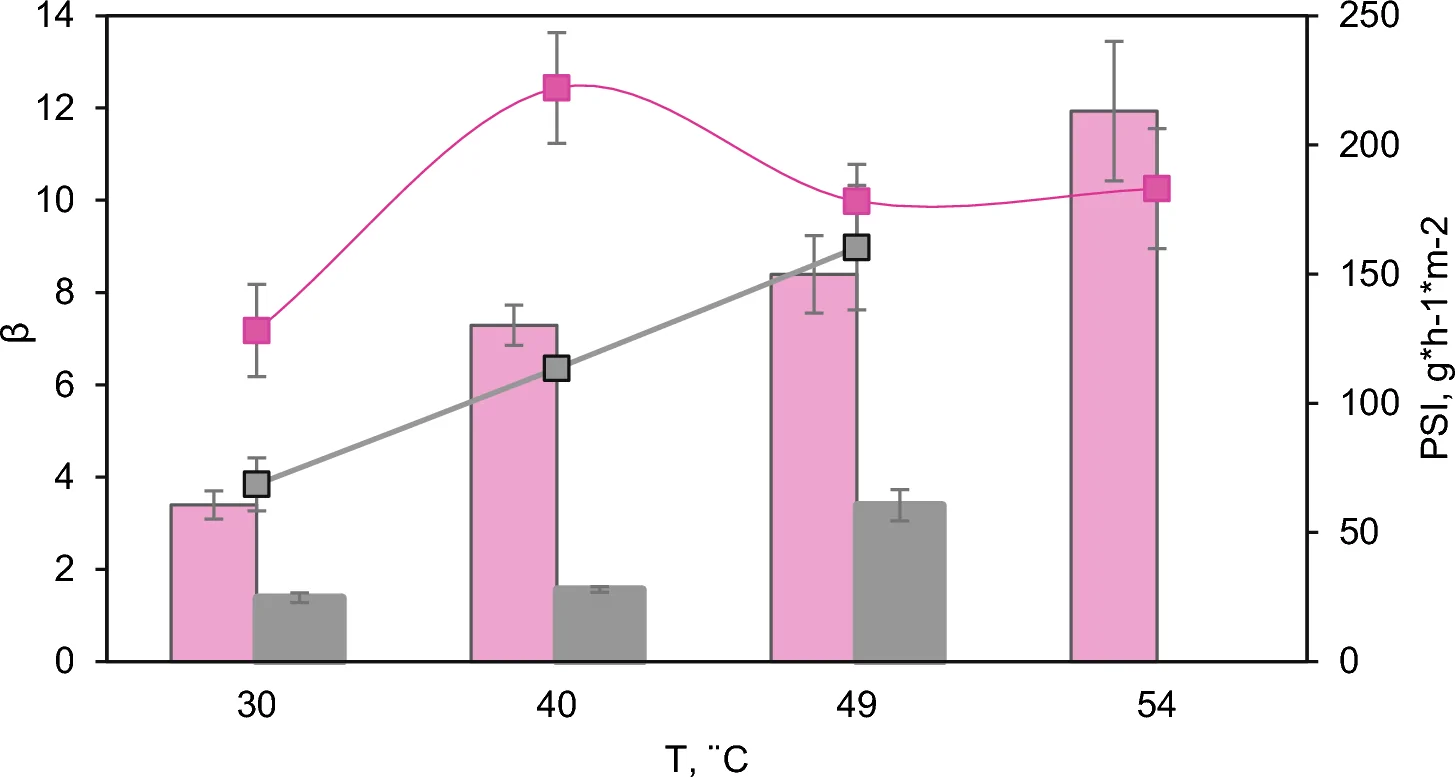
Influence of temperature on β (scatter plot) and PSI (bar chart) for ethanol from 15 g/L ethanol solutions on () the silicone membrane and () the POMS membrane

Tewfik et al. (2022) tested the effect of temperature using a PDMS membrane to pervaporate ethanol from a rice straw fermentation broth containing 7.5% ethanol. The tested range was from 33 to 53 °C, and they quantified the total flux through the membrane, which increased with increasing temperature, from 100 to 1277 g·h^−1^·m^−2^, showing a strong dependence on temperature. In the current study, total flux increased from 507 ± 6 g·h^−1^·m^−2^ at 30 °C to 1247 ± 10 g·h^−1^·m^−2^ at 65 °C. J_EtOH_ increase with temperature was higher than J_total_, indicating higher ethanol selectivity when increasing temperature. To better compare the selectivity of both membranes towards ethanol, β and PSI were evaluated at different temperatures (Fig. 6).

When using the silicone membrane, β increased from 30 to 40 °C and then remained almost constant. In the case of the POMS membrane, β increased with temperature in the studied range. This effect of temperature was also observed in the work carried out using PDMS by Jaimes et al. (2021) as well as in the studies carried out with POMS by (Lazarova et al. 2012), where, at a given concentration in the feed, β increased with temperature. However, the β and PSI values of the silicone membrane were higher at all the studied temperatures when compared to the POMS one. Interestingly, although β remained almost constant above 40 °C with the silicone membrane, PSI continued increasing owing to the substantial increase in permeation flux. The higher the PSI, the more efficient the recovery of the compound of interest by pervaporation is (Alonso-Riaño et al. 2023). Therefore, operating at higher temperatures may be advantageous from a productivity standpoint even when selectivity no longer improves.

Overall, the silicone membrane combined higher permeation flux with higher ethanol selectivity, leading to the highest PSI values over the whole temperature range. Consequently, this membrane and an operating temperature of 54 °C were selected for the subsequent ethanol recovery studies.

#### Effect of ethanol concentration (C)

**Fig. 7:**
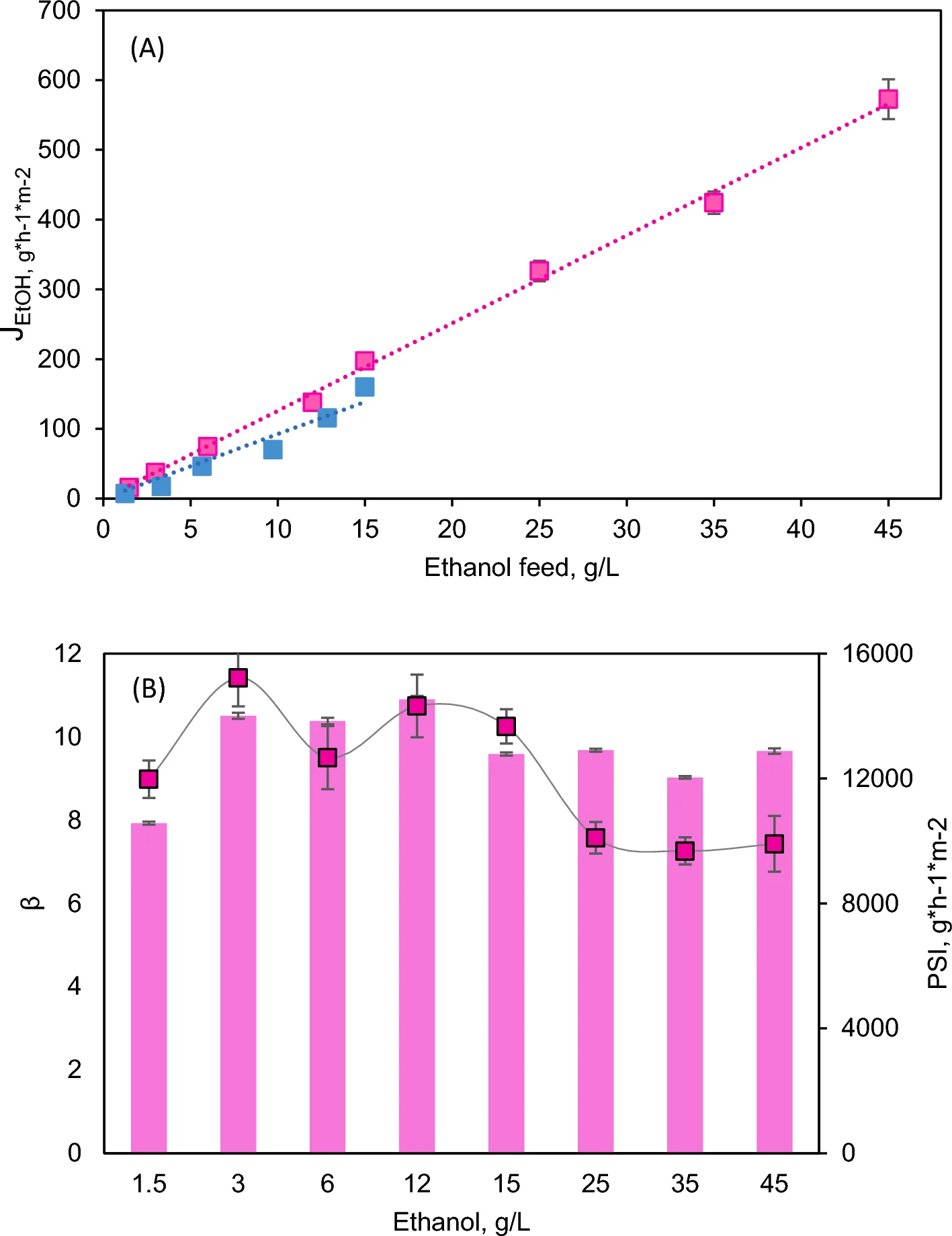
**A** Ethanol permeation flux as a function of ethanol concentration in using the silicone membrane with () binary ethanol/water feeds at 54 °C or () fermentation broth permeation at 51 °C (J_EtOH, binary mixtures_ = 12.58·[EtOH] R^2^ = 0.999, J_EtOH, fermentation broth_ = 9.25·[EtOH] R^2^ = 0.976). **B** Influence of ethanol concentration on the enrichment factor β (scatter plot) and PSI (bar chart) for ethanol in binary ethanol/water mixtures using the silicone membrane at 54 °C

To evaluate the effect of the feed concentration, different ethanol aqueous solutions (1.5, 3, 6, 12, 15, 25, 35 and 45 g/L) were tested using the silicone membrane. The ethanol permeation flux increased almost linearly with ethanol concentration, indicating that the driving force for ethanol transport increased proportionally over the studied concentration range (Fig. 7A).

As indicated by García et al. (2009), as ethanol is a less polar component than water, an increase in the feed concentration leads to greater membrane swelling, resulting in easier movement of ethanol molecules across the silicone membrane due to its hydrophobic character. In contrast, the enrichment factor (β) did not continuously increase with ethanol concentration (Fig. 7B). The highest β values were obtained at intermediate ethanol concentrations (3–15 g/L), whereas a noticeable decrease was observed when the feed concentration reached 25 g/L or higher, stabilizing around β ≈ 7. This behaviour can be attributed to excessive membrane swelling at high ethanol concentrations, which reduces the membrane selectivity by facilitating the transport of water together with ethanol.

This decrease is not as noticeable for PSI because the loss is compensated by an increased flux. Similar results were observed in other studies using different types of polymeric membranes such as POMS (García et al. 2009; Gaykawad et al. 2013) or PDMS (Nakao et al. 1987), indicating that this behaviour is characteristic of hydrophobic polymeric membranes rather than a specific feature of the silicone membrane used in this work.

### Ethanol recovery from fermentation broth (Strategy 1)

**Fig. 8:**
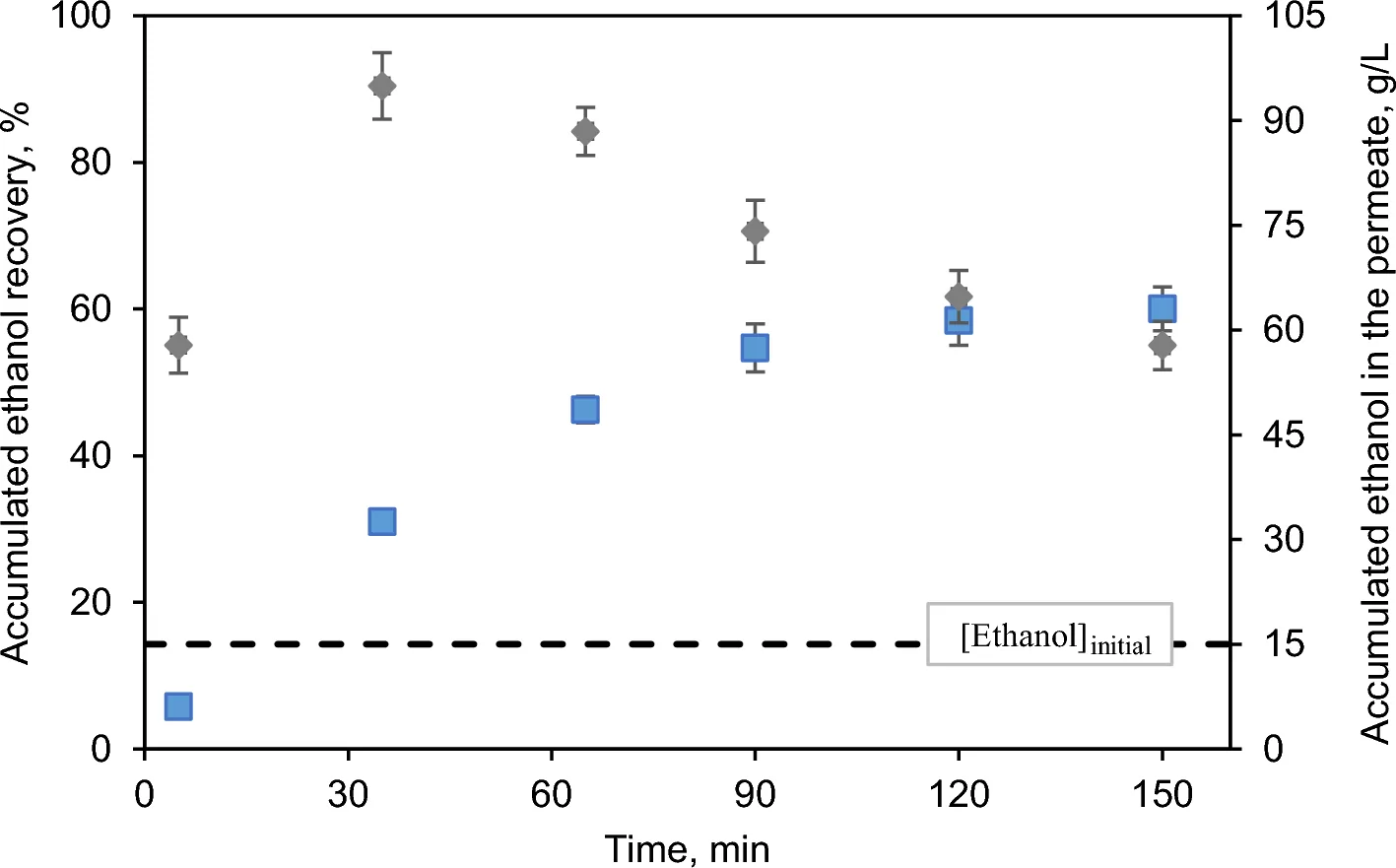
Time evolution of the () accumulated ethanol recovery (%) and () purity (g/L) of recovered ethanol during pervaporation from the fermentation broth using the silicone membrane

To assess membrane performance under realistic operating conditions, the fermentation broth obtained after alcoholic fermentation was also subjected to pervaporation. As shown in Fig. 7A, the ethanol permeation flux obtained with the fermentation broth followed the same increasing trend as that observed for binary ethanol/water solutions, although the flux was approximately 26% lower at similar ethanol concentrations (15 g/L) and operating temperatures (51 ± 2 °C for the fermentation broth and 54 ± 2 °C for the binary solution). Furthermore, 60 ± 3% of the ethanol present in the fermentation broth was recovered, reaching a final ethanol concentration of 58 ± 4 g/L in the permeate (Fig. 8). The lower permeation performance compared with binary mixtures is attributed to the greater complexity of the fermentation broth, where the presence of non-volatile compounds may modify ethanol sorption and increase mass transfer resistance. A similar reduction in ethanol flux (17–20%) when processing fermentation broths instead of synthetic ethanol solutions has also been reported for PDMS membranes (Nakao et al. 1987).

Therefore, the ethanol concentration obtained after fermentation (≈15 g/L) lies within the concentration range where the membrane still exhibits high selectivity and PSI values, confirming the suitability of the selected membrane for coupling with the fermentation process.

### Simultaneous fermentation and pervaporation (SFP) (Strategy 2)

Following the optimization of the pervaporation conditions, the fermentation and ethanol recovery steps were integrated into a simultaneous fermentation–pervaporation (SFP) process to evaluate whether continuous ethanol removal could enhance the overall process performance while simultaneously concentrating the product.

After 4 h of fermentation (yeast adaptation period) in the pervaporation tank, the simultaneous pervaporation was started. The SFP was carried out at the recommended fermentation temperature (28 °C) to ensure the survival of the microorganism, and the fermentation broth was prepared in the same conditions as the fermentation in Strategy 1 (32 g/L initial glucose concentration).

**Fig. 9:**
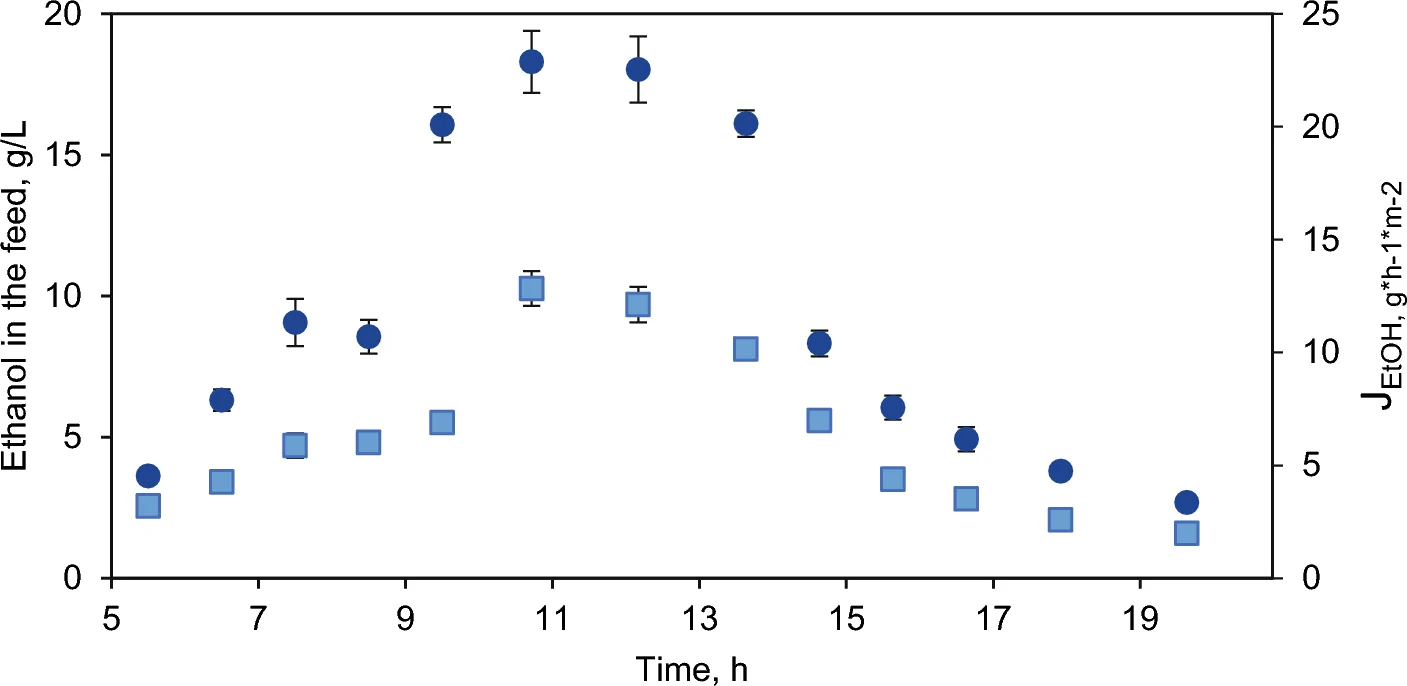
Time evolution of simultaneous fermentation and pervaporation at 28 °C using the silicone membrane. The ethanol permeate flux () is expressed as g /h*m^2^ and the ethanol concentration in the feed () as g/L broth

As shown in Fig. 9, ethanol formation showed an increasing trend until 11 h of fermentation, reaching a concentration of 10.3 ± 0.6 g/L. In this period, the ethanol production rate by fermentation was higher than ethanol separation rate by pervaporation. Following this time, the ethanol concentration in the feed started to decrease, and therefore, the rate of ethanol separation was higher than the rate of ethanol formation.

Comparing these results with Fig. 4, it was observed that the kinetics of ethanol formation were similar to the sequential fermentation process up to 11 h, presenting the following ethanol concentrations: sequential fermentation 2.1 g/L and 4.2 g/L at 6 h and 8 h, respectively; SFP 2.6 g/L and 4.7 g/L at 6 h and 8 h, respectively. For times above 11 h, it was not possible to compare the ethanol formation trend because more ethanol was permeated than formed. Once finalized, the pervaporation yield of the SPF strategy was 97 ± 6, while this value was 60 ± 3 (Fig. 8) in the sequential strategy at the same temperature. When also taking the fermentation step into account, the overall yield of the cascade final stages was 55 ± 3% in Strategy 1, while it increased to 70 ± 4 in Strategy 2.

The maximum ethanol flux was 22.9 g·h^−1^·m^−2^, reached at 11 h, coinciding with the maximum ethanol concentration in the feed (10.3 g/L). Compared to the ethanol flux obtained from the sequential strategy, the ethanol permeate flux was lower than in the previous fermentation permeate experiment with similar ethanol concentration (J_EtOH_ _(9.73 g/L)_ = 70 g·h^−1^·m^−2^) due to the different operating temperatures (Fig. 7A). If the SFP flux (11 h, 10.3 g/L ethanol) is compared at a similar temperature, the flux of the 15 g/L ethanol solution experiment at 30 °C, was more similar: J_EtOH_ _(15 g/L)_ = 54.8 g·h^−1^·m^−2^ versus J_SFP_ = 22.9 g·h^−1^·m^−2^, although still lower. This difference in flux may be due to the previously mentioned effect of the presence of other components in the fermentation broth, such as the yeast, sugars or the supplementation solution, which may interfere with the flow through the membrane; as well as to the difference in ethanol concentration in the feed (SFP = 10.3 g/L; Ethanol solution = 15 g/L).

As indicated by Nakao et al. (1987), biomass could accumulate on the membrane surface reducing the effective pervaporation area and resulting in a lower flux. In fact, in this study it was observed that the membrane changed color and yeast residues were accumulated in the pervaporation cell. Additionally, these authors indicated that the inorganic salts added as nutrients are not completely consumed during fermentation and accumulate in the fermentation broth (Nakao et al. 1987).

More recently, Gaykawad et al. (2013) performed a sequential barley straw hydrolysate fermentation to bioethanol, and then recovered the ethanol through PV using a POMS membrane. They studied membrane fouling in depth, and observed a significant difference when working with standard solutions or biomass fermentation broth. In the case of the broth, they observed a total flux decrease of 20% and determined that with their working conditions, the membrane fouling was irreversible. (Gaykawad et al. 2013). These observations suggest that membrane fouling may become one of the limiting factors during prolonged SFP operation. In the current study, water at 30 °C was used to test membrane fouling by studying the flux though the membrane prior to pervaporation or SFP and comparing it to the flux after those experiments. In the case of PV in the sequential strategy, the total permeate flux, *J*_*total*_ (Eq. 3) decreased from 344 ± 7 to 341 ± 9 g∙h^−1^.m^−2^ after the PV of the fermentation broth, showing no significant difference. In the case of the SFP at 28 °C, *J*_*total*_ decreased in a higher extent, from 344 ± 7 to 332 ± 10 g·h^−1^∙m^−2^, but the change was still no significant.

Additionally, *J*_*total*_ was quantified during all the PV experiments, to better asses membrane performance along the different strategies. Initial and final *J*_*total*_ of each experiment are included in Table 2, as well as *J*_*total*_ reduction.

**Table 2:** *J*_*total*_ variation during PV in Strategy 1 and SFP at 28 and 38 °C

	Strategy 1 (51 °C)	Strategy 2 (28 °C)		Strategy 2 (38 °C)
Initial *J*_*total*_	1038 ± 54	343 ± 15	343 ± 14	
Final *J*_*total*_	627 ± 16	294 ± 15	351 ± 15	
PV time (h)	2.5	18	18	
*J*_*total*_ reduction (%)	40 ± 2	14 ± 1	0.29 ± 0.01	

As it can be seen, the highest *J*_*total*_ reduction was observed in Strategy 1 (sequential fermentation and pervaporation), with a 40 ± 2% after only 2 h, from 1038 to 627 g∙h^−1^·m^−2^. However, the testing with water indicated no significant changes in the membrane *J*_*total*_ after PV, indicating that although *J*_*total*_ decreases during the PV study, and visual residues could be accumulated in the membrane, the general membrane performance was not affected and its total flux was recovered. The same behaviour was observed in the membrane used in Strategy 2 at 28 °C, although in this case *J*_*total*_ reduction during the PV was 14 ± 1% after a longer period of 18 h.

Zabihi and Esfahanian (2024) compared cell density growth during a batch fermentation of sugarcane bagasse sugars hydrolysate and during an integration of batch fermentation and PV using a PDMS-CNT/Tetrazole at 32 °C. They observed that after 25 h, cell concentration was 1.5 g/L and 0.5 g/L, respectively, and they attributed this effect to the continuous ethanol removal from the reaction vessel (Zabihi and Esfahanian 2024). Although cell growth was not reported in the SFP experiments, it is possible that this effect in cell density also took place, improving pervaporation performance, while reducing membrane fouling as well (Table 2).

If the whole process is compared for both strategies at 28 °C, and assuming the same previous steps before fermentation, there is interesting additional information to compare. As described in Table S1, starting from 1 kg of dry corn stover, by the beginning of the sequential fermentation or the SFP at 28 °C, 0.21 ± 0.01 kg of glucose would be available for ethanol production in a total volume of 6.5 ± 0.4 L. As a result of sequential fermentation at 28 °C followed by PV at 51 °C, the amount of recovered ethanol would be 0.058 ± 0.004 kg of ethanol in the permeate, which would have 1 L volume, and therefore an ethanol concentration of 58 ± 4 g/L. Regarding the SFP at 28 °C, the final ethanol concentration would be lower, corresponding to 36 g/L, in a recovered permeate volume of 2.1 ± 0.1 L. This process would produce 0.075 ± 0.005 kg of ethanol, a higher amount than in the sequential strategy, coinciding with the higher previously described overall yield. The drawback of the SFP strategy at this temperature is that there would be a higher final volume to manage and recover ethanol from.

#### Optimization of SFP: effect of fermentation temperature on SFP performance

**Fig. 10:**
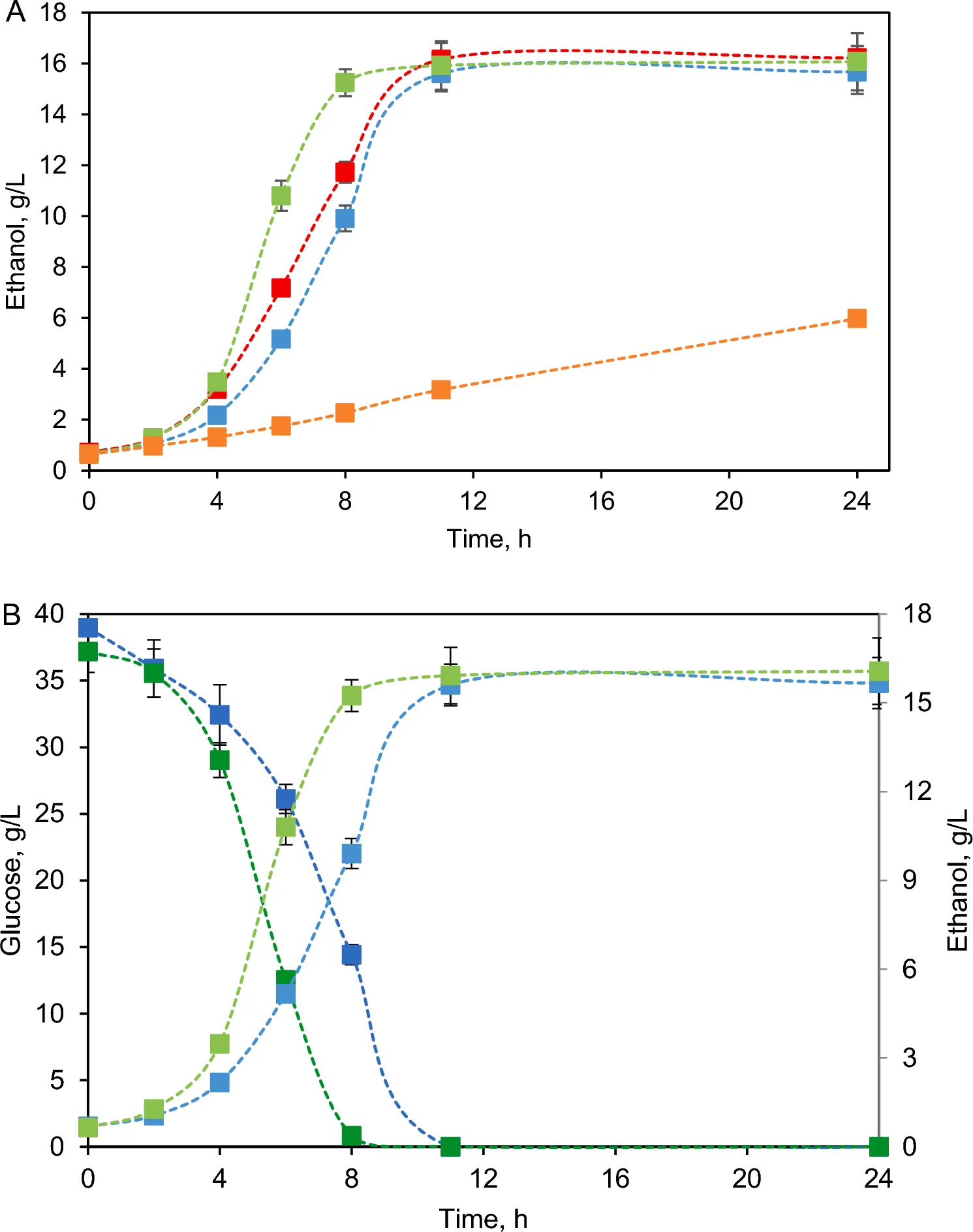
**A** Time course of ethanol concentrations (g/L) in alcoholic fermentation from 40 g/L glucose solutions at 28 °C (), 33 °C (), 38 °C () and 43 °C (). **B** Time course of glucose and ethanol concentrations (g/L) in alcoholic fermentation at the recommended (28 °C,  and  respectively) and optimal (38 °C,  and  respectively) temperatures

Since pervaporation benefits from increasing temperature whereas yeast growth has an optimum at lower temperatures, the influence of fermentation temperature on the integrated process was evaluated. Four fermentation experiments were carried out at 28, 33, 38 and 43 °C using a 40 g/L glucose solution (Fig. 10A).

Ethanol production was similar in the lower temperature tests. However, at 43 °C, yeast viability was compromised and fermentation significantly slowed down. At 38 °C, ethanol formation during the initial stages (4–8 h) was even faster than at the recommended temperature of 28 °C, reaching 15.3 ± 0.7 g/L ethanol in just 8 h.

This observation coincided with faster glucose consumption at 38 °C than at 28 °C (Fig. 10B), favouring the efficiency of the process. Based on these results, 38 °C was selected for the subsequent SFP experiments, as it represented the best compromise between fermentation performance and pervaporation efficiency.

#### SFP at higher T

**Fig. 11:**
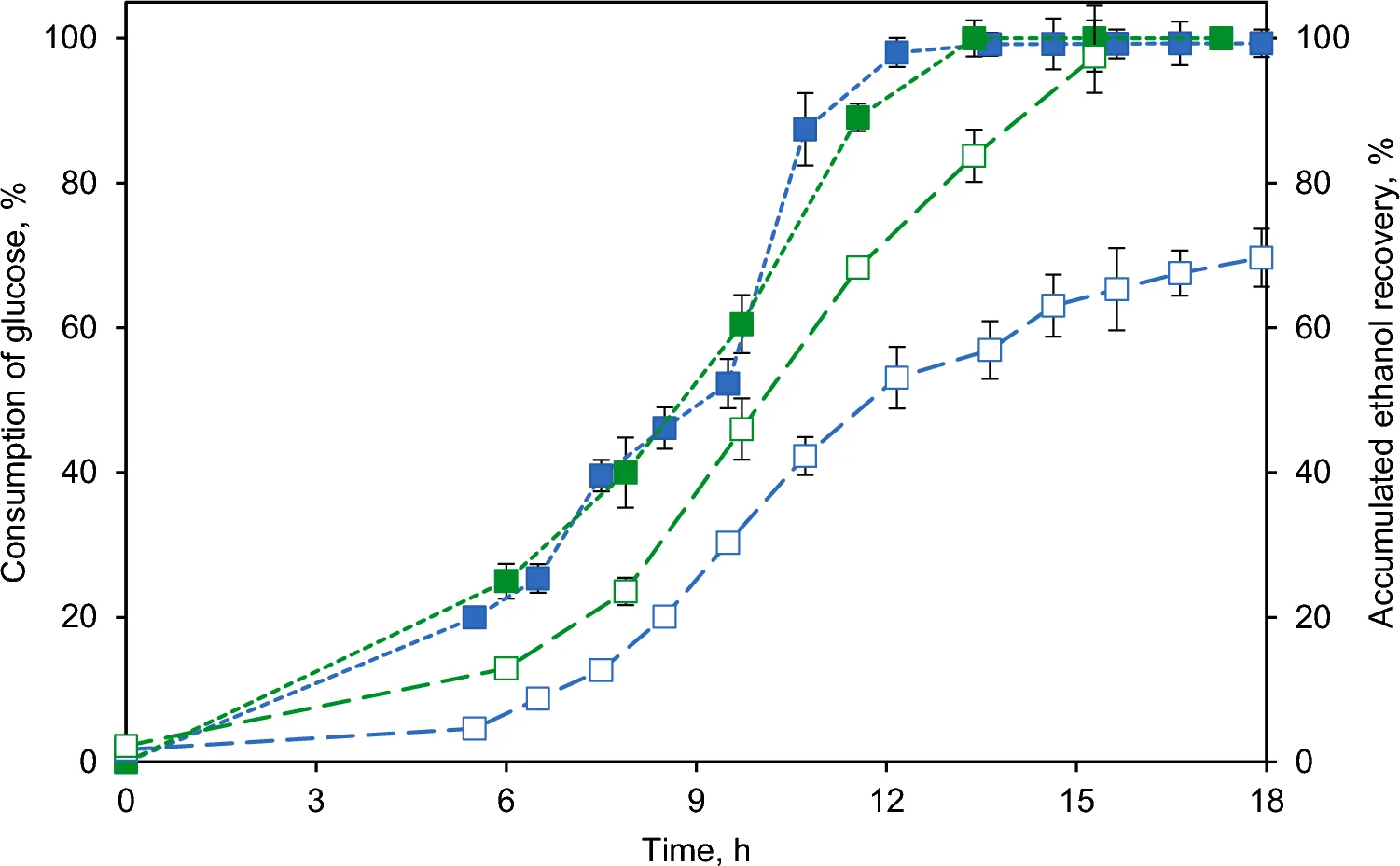
Glucose consumption in the broth (, ) and the accumulated ethanol in the permeate (, ) during SFP with the silicone membrane at 28 °C (, ) and 38 °C (, )

Simultaneous fermentation and pervaporation using the HE corn stover subW residue hydrolysate was carried out at the selected temperature of 38 °C, and compared to the one at 28 °C, the fermentation recommended temperature (Fig. 11).

Glucose consumption was similar at both tested temperatures, being depleted between 12 and 14 h, which confirms that increasing the temperature in the SFP process does not compromise the fermentation, as shown with pure glucose solutions (Fig. 10A).

The maximum ethanol concentration in the feed was the same in both processes (10 g/L after 11–12 h), but the ethanol flux at 38 °C was much higher (62 g/h*m^2^) than at 28 °C (23 g/h*m^2^), allowing for a greater ethanol recovery.

Operating the SFP process at 38 °C substantially improved ethanol recovery, allowing 98 ± 7% of the produced ethanol to be recovered within 15.3 h, compared to only 70 ± 4% at 28 °C. Temperature not only favored the pervaporation performance, but also the fermentation one, as observed in Fig. 10B. Using the SFP at 38 °C outperformed the other tested approaches, showing a overall yield of 97 ± 8%, while the same process at 28 °C yielded 70 ± 4% and the sequential strategy only achieved 55 ± 3%.

**Fig. 12:**
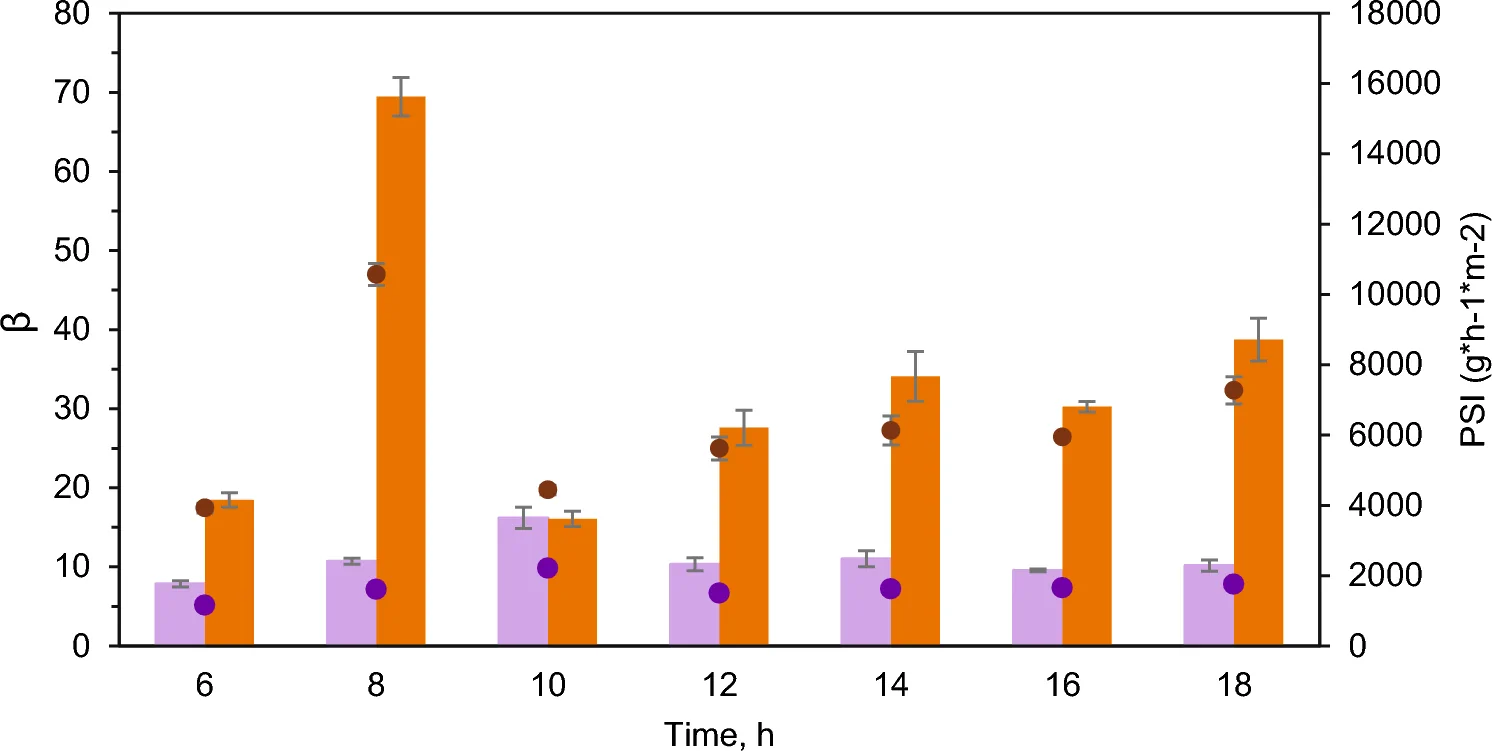
Enrichment factor, β (scatter plot) and PSI (bar chart) during SFP at 28 °C (, ) or 38 °C (, )

The favouring conditions for PV provided by the higher temperature could also be observed in other pervaporation performance indicators (Fig. 12).

Regarding the enrichment factor (β), which indicates the ratio of ethanol concentration in the permeation to the feed, it was higher at 38 °C than at 28 °C at all times, showing a maintained improved PV yield and the best enrichment factor among all experiments at time = 8 h. PSI showed a similar behaviour, showing the highest value after 8 h of PV. The provided data cannot be directly compared to the PV process of the sequential strategy, since in SFP there is ethanol formation at the same time of ethanol removal in the feed, while in Strategy 1 there is only ethanol removal from the feed.

As SFP is a novel approach, no similar process is available in literature to properly compare. For example, Fan et al. (2014) performed a continuous closed-circulating system, which consisted in first, a 24 h fermentation at 35 °C, and then the recirculation of the fermentation broth directly to a pervaporation system. Therefore, this process would be more similar to the sequential strategy of this study. They later pervaporated the broth up to 192 h or 264 h (Fan et al. 2014). No comparable PV performance parameters was provided by the authors, but only the reduction time factor achieved in the current SFP at 38 °C with an overall yield of 97 ± 8% makes of the present Strategy an improved alternative.

When looking to the process comparison in Table S1, starting from 1 kg of raw corn stover, SFP at 38 °C showed the best results. The relatively small volume (1.4 L) of permeate would have an ethanol concentration of 71 ± 5 g/L, accounting a total of 0.104 ± 0.007 kg of ethanol. This amount is significantly higher than the ethanol recovered from 1 kg of corn stover after the completion of the other two strategies. Therefore, SFP at 38 °C was the best strategy under the current set up and tested conditions for ethanol recovery from raw corn stover.

Furthermore, *J*_*total*_ reduction was much smaller during Strategy 2 PV at the highest temperature (Table), which was 0.29 ± 0.01%. The mentioned PV testing conditions did not decrease total flux through the membrane even when it was used with fermentation broths and long testing periods, once again showing that SFP at 38 °C is the best Strategy and that allows for continuous and long-term operation conditions.

A moderate increase in fermentation temperature can simultaneously intensify the pervaporation step without compromising yeast activity, providing an effective operating window for the integration of both processes.

Overall, the proposed simultaneous fermentation–pervaporation strategy demonstrates that process integration can intensify bioethanol production by enhancing ethanol recovery and simplifying downstream processing, highlighting its potential for the sustainable valorization of lignocellulosic biomass.

## Conclusions

This study demonstrates a cascade biorefinery for corn stover valorization by integrating subcritical water pretreatment, enzymatic hydrolysis, fermentation, and pervaporation for bioethanol production and recovery. Subcritical water pretreatment effectively solubilized hemicellulose and increased cellulose accessibility, resulting in approximately 80% glucose yield during enzymatic hydrolysis. Sequential fermentation of the hydrolysate achieved a maximum ethanol yield of 91% after 15 h, exceeding many literature values. Pervaporation with a silicone membrane provided efficient ethanol recovery with higher flux and selectivity than a POMS membrane, with optimal performance at 54 °C. Simultaneous fermentation and pervaporation enhanced process efficiency through continuous ethanol removal, and best results were found when SFP temperature was optimized. At 38 °C, glucose consumption and ethanol production were accelerated, enabling recovery of 98% of produced ethanol within 15.3 h and reaching a combined process overall yield of 97%, outperforming the sequential strategy, and SFP at 28 °C, with 55 and 70% overall yields, respectively. These results confirm the feasibility of integrating thermochemical, biochemical, and membrane-based operations for efficient lignocellulosic bioethanol production. Further optimization of individual steps and their integration could establish an economically viable and sustainable biorefinery concept for corn stover valorization.

## Supplementary Information

Additional file1 (DOCX 16 kb)

## Data Availability

The datasets used and/or analysed during the current study are available from the corresponding author on reasonable request.
