# Supplementary material for: Integrated simultaneous fermentation–pervaporation for intensified bioethanol production and recovery in a corn stover cascade biorefinery
Source: Bioresour Bioprocess. 2026 Nov 7;13(1):164. doi: 10.1186/s40643-026-01152-y (PMC13744582; doi:10.1186/s40643-026-01152-y)
Supplement: Supplementary file 1 [file 40643_2026_1152_MOESM1_ESM.docx]

**Table S1**. Transformation of 1 kg of dry corn stover through the cascade steps evaluated in the manuscript, indicating the desired product after each step, its yield, concentration and amount, as well as if it is in the liquid or solid fraction, and the quantity of those fractions.

| Cascade step | Stage | Product | Product yield (%) | Product concentration (g/L) | Product amount (kg) | Liquid (L) | Solid (kg) |
| --- | --- | --- | --- | --- | --- | --- | --- |
| 1. SubW | Initial (corn stover + water) |  |  |  |  | 20 | 1 |
|  | After subW treatment | Furfural | 29.1 ± 1.2 | 3.2 ± 0.1 | 0.055 ± 0.002 | 17.2 ± 1.1 | 0.44 ± 0.03 |
| 2. HE | Initial (subW solid + HE mix) | Cellulose |  | 52.7 ± 3.6 | 0.29 ± 0.02 | 5.5 ± 0.4 | 0.44 ± 0.03 |
|  | After HE treatment | Glucose | 80 ± 3 | 42 ± 3 | 0.21 ± 0.01 | 5.3 ± 0.3 | 0.19 ± 0.01 |
| 3. Fermentation at 28 °C | Initial (HE liquid + F mix) | Glucose |  | 32 ± 3 | 0.21 ± 0.01 | 6.5 ± 0.4 | - |
|  | After fermentation | Ethanol | 91 ± 1 | 14.9 ± 0.8 | 0.097 ± 0.007 | 6.5 ± 0.4 | - |
| 4. Pervaporation at 51 °C | Initial (F liquid) | Ethanol |  | 14.9 ± 0.8 | 0.097 ± 0.007 | 6.5 ± 0.4 | - |
|  | After PV | Ethanol | 60 ± 3 | 58 ± 4 | 0.058 ± 0.004 | 1.00 ± 0.05 | - |
| 5. SFP at 28 °C | Initial (HE liquid + F mix) | Glucose |  | 32 ± 3 | 0.21 ± 0.01 | 6.5 ± 0.4 | - |
|  | After PV | Ethanol | 97 ± 6 | 36 ± 2 | 0.075 ± 0.005 | 2.1 ± 0.1 | - |
| 6. SFP at 38 °C | Initial (HE liquid + F mix) | Glucose |  | 32 ± 3 | 0.21 ± 0.01 | 6.5 ± 0.4 | - |
|  | After PV | Ethanol | 98 ± 7 | 71 ± 5 | 0.104 ± 0.007 | 1.4 ± 0.1 | - |

subW: Subcritical water; HE: Enzymatic hydrolysis; F: Fermentation; PV: Pervaporation; SFP: Simultaneous fermentation and pervaporation

Note: calculations have been performed according to the products yield and concentrations given along the manuscript in each step. In the case of SFP, the ethanol yield indicates the ethanol recovery yield after its PV, and its concentration and amount in the permeate.
